# Social engagement within the facility increased life expectancy in nursing home residents: a follow-up study

**DOI:** 10.1186/s12877-020-01876-2

**Published:** 2020-11-18

**Authors:** Roberto Pastor-Barriuso, Alicia Padrón-Monedero, Lina M. Parra-Ramírez, Fernando J. García López, Javier Damián

**Affiliations:** 1 National Center for Epidemiology, Institute of Health Carlos III, Av/ Monforte de Lemos 5, 28029 Madrid, Spain; 2 Consortium for Biomedical Research in Epidemiology and Public Health (CIBERESP), Madrid, Spain; 3 Department of Preventive Medicine and Public Health, Autonomous University of Madrid/ IdiPAZ, Madrid, Spain; 4 Consortium for Biomedical Research in Neurodegenerative Diseases (CIBERNED), Madrid, Spain; 5 Preventive Medicine Department, Puerta de Hierro Majadahonda University Hospital, Madrid, Spain

**Keywords:** Cohort study, Inverse probability weighting, Mortality, Nursing homes, Social engagement.S

## Abstract

**Background:**

Social engagement (SE) has been consistently shown to improve survival among community-dwelling older people, but the evidence in nursing home residents is inconclusive and prone to short-term reverse causation and confounding by major health determinants. Our main objective was to study the potential causal effect of within-the-facility social engagement (SE) on long-term all-cause mortality in care home residents.

**Methods:**

A representative cohort of 382 nursing home residents in Madrid without severe physical and cognitive impairments at baseline was followed up for 10-year all-cause mortality. Standardized mortality curves for residents with low/null, moderate, and high levels of SE at baseline were estimated using Kaplan-Meier methods and spline-based survival models with inverse probability of exposure weights conditional on baseline sociodemographic characteristics, facility features, comorbidity, and disability. Standardized 5-year mortality risks and median survival times were compared across levels of SE.

**Results:**

The baseline prevalences of low/null, moderate, and high SE were 36, 44, and 20%, respectively. Compared with residents with low/null SE at baseline, the standardized differences (95% confidence intervals) in 5-year mortality risk were − 2.3% (− 14.6 to 10.0%) for moderately engaged residents and − 18.4% (− 33.8 to − 2.9%) for highly engaged residents. The median survival time increased by 0.4 (− 1.4 to 2.2) and 3.0 (0.8 to 5.2) years, respectively.

**Conclusion:**

Residents with high SE within the nursing home had an 18% lower 5-year mortality risk and a 3-year increase in their median survival, as compared with residents with similar health determinants but low/null SE. The development of adequate tailored intervention programs, addressed to increase SE in nursing home residents, could improve their long-term survival, in addition to expected gains in quality of life.

**Supplementary Information:**

The online version contains supplementary material available at 10.1186/s12877-020-01876-2.

## Background

The World Health Organization recommends the promotion of active ageing as a way to enhance not only the physical and mental health status of older people, but also their active participation in society according to their needs, desires, and capacities [1]. Social engagement (SE), according to Herzog et al., has been conceptualized as the activities that the person undertakes within the framework of its social environment [2] including social, productive, helping, educational/intellectual and leisure activities, that often overlap [2] and can have blurred boundaries between each other. SE tends to decrease in older age [2], and there is evidence that – irrespective of other health determinants – lack of emotional support and loneliness are independently associated with increased risk for long-term all-cause mortality among older adults living in the community [3, 4] while SE has a beneficial influence on survival [2]. In a residential setting, the subsequent loss of prior social networks in the community may further increase the likelihood of social isolation. Hence, meaningful interactions between residents and active participation in organized facility activities could play an important role in maintaining SE [5]. However, the direct evidence linking SE to longer survival in nursing home residents is limited and inconclusive, as some cohort studies [6, 7] had short 1-year follow-up periods that could make them difficult to rule out a possible reverse causation bias; low levels of SE may reflect undetected frailty conditions near death. Furthermore, other long-term cohorts lacked representative samples of institutionalized older people [8, 9], or failed to properly adjust for potential confounding from the main common independent determinants of SE and mortality [10].

Therefore, this study aims to analyze the potential causal relation between SE in nursing homes and long-term all-cause mortality in a representative sample of nursing home residents in Madrid, by using inverse probability of exposure weighting to adjust for differences in other health determinants.

## Methods

We followed equivalent procedures as described elsewhere [11], yet with additional methods coherent with the objectives of the present work.

### Study population

This study used mortality follow-up data from a survey carried out between 1998 and 1999 in a representative sample of people 65 years or older living in care homes in Madrid, Spain. The sample was assembled by means of a stratified cluster sampling, by selecting 25 public/subsidized and 30 private facilities with probability proportional to their size, and then randomly sampling 10 men and 10 women from each selected public/subsidized facility, and 5 men and 5 women in private institutions. Of the 800 sample residents, 85 subjects refused to participate (response rate 89%) and 39 of these were randomly replaced with residents of the same facility and sex, for a total of 754 participants in the baseline survey. Residents in public/subsidized facilities and men were oversampled; accordingly, sampling weights were computed for study subjects as the inverse of their probability of selection. Subjects were eligible for the present study if they were residents of nursing homes in Madrid, Spain, aged ≥65 years, and did not suffer from severe physical or cognitive impairments at baseline (severe or total functional dependency, severe cognitive impairment, physician’s diagnosis of dementia, or behavioral problems).

### Baseline data collection

Trained geriatricians and residents in geriatrics interviewed the residents, their main caregivers, the facility staff, and the facility physicians to gather information on sociodemographic data, facility characteristics, SE within the facility, external contact with family and friends, morbidity, functional dependency, cognitive status, and behavioral problems.

By interviewing residents (or next of kin) we obtained information about their age, sex, education, and marital status. By interviewing the facility staff, we obtained information about facility size, caregivers assigned to residents, and length of stay in the facility.

Residents’ SE levels within the nursing home were determined according to each resident’s degree of interaction with other residents, and to their level of active participation in the activities of the facility. To this purpose, study subjects (86%) or their main caregivers (14%) were asked the following question: “To what extent do you/does the resident interact with other residents in the institution: 1. A lot; actively engages in the facility activities; 2. Sufficiently, normal; 3. Hardly; 4. Not at all”. Participants were classified into three levels of SE according to the option selected: high (option 1), moderate SE (option 2), and low/null (options 3 and 4). Information about frequency of external contacts was also obtained from the study subjects or their main caregivers, and classified as monthly or less, weekly, or daily.

We ascertained chronic diseases by interviewing facility physicians (or nurses for 8% of residents) with access to medical histories. These included chronic obstructive pulmonary disease, ischemic heart disease, heart failure, arrhythmias, peripheral arterial disease, stroke, high blood pressure, diabetes, anemia, Alzheimer’s disease, other dementias, Parkinson’s disease, epilepsy, depression, anxiety, arthritis, and cancer. Dementia was defined as a physician’s diagnosis of Alzheimer’s disease or other dementias. The number of these conditions, not including dementia, was registered and categorized into 0–1, 2–3, and ≥ 4 diseases.

Functional dependency in performing activities of daily living was evaluated by interviewing residents or their main caregivers using a Barthel index version [12]. According to this version [12], residents were categorized as independent (100 points), mild dependency (91–99 points), moderate dependency (61–90 points), and severe or total dependency (0–60 points).

Cognitive status was evaluated with the Pfeiffer’s Short Portable Mental Status Questionnaire (0–10 errors) [13], which was adapted to the institutional setting, and the Minimum Data Set Cognition Scale (0–10 points) [14], which obtained an assessment from the main caregivers based on particular Minimum Data Set questions. Severe cognitive impairment was defined as 8 or more education-adjusted errors in the Short Portable Mental Status Questionnaire or 9 or more points on the Minimum Data Set Cognition Scale. Residents with behavioral problems related to verbal, physical abuse, or inappropriate/disruptive behavior during the previous week were identified through the corresponding Minimum Data Set questions answered by caregivers.

### Mortality ascertainment during follow-up

All-cause mortality was ascertained up to September 2013. We used two separate sources to double check the mortality ascertainment of nursing home residents. We first asked participating facilities about residents’ vital status and, for this specific study, we also requested access to the Spanish National Death Index, which comprises all deaths recorded in Spain [15], and ascertained via computerized linkage if any of study participants appeared as deceased. Follow-up time for each resident was computed from their 1998–1999 baseline interview until death, unless they were censored at 10 years of follow-up or age 105 years, whichever occurred first.

### Statistical analysis

The cumulative all-cause mortality curves for each baseline level of SE within the nursing home (low/null, moderate, or high) were standardized to the weighted distribution of baseline confounders in the overall institutionalized population by using inverse probability weighting. We first fitted a sampling-weighted polytomous logistic model to estimate each resident’s probability of being in their own level of SE given the observed confounders. Standardization weights were computed as the inverse of these conditional probabilities, and were further rescaled by the sampling-weighted proportions in each level of SE to reduce variability of weights and avoid influential observations with extreme weights [16]. We then assigned combined weights to residents as the product of the sampling weights and the standardization weights to correct for both selection bias and confounding [17].

Three increasingly comprehensive sets of baseline confounders were included in the polytomous logistic model for baseline SE. The first model included age (65–74, 75–79, 80–84, 85–89, or ≥ 90 years), sex (women or men), education (less than primary; primary; or secondary or more), and marital status (married, single, or widowed/divorced). The second model additionally controlled for facility ownership (public/subsidized or private), facility size (< 100, 100–299, or ≥ 300 beds), length of stay (0–1, 2–4, or ≥ 5 years), assigned caregiver (yes or no), and frequency of external visits (monthly or less, weekly, or daily). The third model further adjusted for baseline multimorbidity and disability, including number of diseases (0–1, 2–3, or ≥ 4) and functional dependency (no, mild, or moderate). The mean (range) combined weights, taking into account both sampling and standardization based on these models, were 0.99 (0.18–3.25), 0.99 (0.14–4.12), and 0.98 (0.13–3.77) for the first, second and third model, respectively (Supplementary Figure 1). This weighting procedure provided reliable standardization, as can be appreciated by the fact that the weighted distributions of confounders were almost equal across the different levels of SE, and also narrowly matched their sampling-weighted distributions in the entire institutionalized population (Supplementary Table 1).

For estimating mortality risks, we used Kaplan-Meier methods and flexible parametric survival models using splines [18], weighted by combined weights and stratified by level of SE to obtain nonparametric and smooth estimates of the cumulative mortality curves that would have been observed in the overall institutionalized population had every resident been in each level of SE [19]. For models based on splines, stratum-specific log cumulative hazards were modelled as distinct natural cubic splines of log time with two internal knots at the 33th and 67th percentiles [18]. We used these weighted spline-based survival models to compute standardized differences in cumulative mortality at 2, 5, and 10 years of follow-up, as well as standardized differences in median survival times, for moderate and high SE compared with low/null SE. The 95% confidence intervals (CIs) were derived by applying delta methods on robust standard errors of spline coefficients.

We assessed potential modifications in risk differences among relevant subgroups of residents based on baseline age, sex, facility ownership, facility size, and baseline functional dependency by using weighted spline-based survival models stratified by baseline level of SE and participant subgroup. Weights for subgroup analyses were calculated as the product of sampling weights and subgroup-specific standardization weights, which allowed to standardize cumulative mortality curves for each SE level and resident subgroup to the weighted distribution of confounders in the entire resident subgroup [16]. We estimated standardized differences in 5-year cumulative mortality and their 95% CIs within each subgroup, and tested for heterogeneity across subgroups by using joint Wald tests. Statistical analyses were run with the *stpm* command in Stata, version 14 (Stata Corp., College Station, Texas) and graphics were produced in R, version 4 (R Foundation for Statistical Computing, Vienna, Austria).

## Results

Among the 754 residents in the baseline survey, 32 (4.2%) with missing data in one or more baseline covariates and 55 (7.3%) with indefinite vital status at the end of follow-up were excluded. We also excluded 270 residents (35.8%) with severe or total functional dependency (179 residents), severe cognitive impairment (12 residents), physician’s diagnosis of dementia (69 residents), or behavioral problems at baseline (10 residents), since it is conceivable that people with these conditions would not be candidates for an eventual study of an intervention program to promote SE. Additionally 15 participants (2.0%) who had stays shorter than 60 days were excluded, so as to exclude most short-stay rehabilitation patients. Thus, the final cohort included 382 long-term residents without severe physical or cognitive impairments. There were 124 (36.0%) with low/null SE, 179 (44.2%) moderate SE, and 79 residents (19.8%) with high levels of SE at baseline. There were 61 residents (with a sampling-weighted percentage of 15.7%) ranging in age from 65 to 74 years, 70 (16.4%) ranging from 75 to 79 years, 102 (25.6%) ranging from 80 to 84 years, 81 (23.8%) ranging from 85 to 89 years and 68 (18.5%) with 90 or more years old at baseline. As a result of our sample selection methodology, the women were 200 (with a sampling-weighted percentage of 74.4%) of our participants and men 182 (25.6%). The educational level was less than primary for 188 (45.3%) of the participants, primary for 143 (40.2%) and secondary or more for 51 (14.6%). The marital status was married for 68 (14.2%) of the participants, single for 113 (33.7%) and widowed/divorced for 201 (52.1%) (Table 1). Highly engaged subjects were more likely to be younger, men, currently or previously married, residents in medium-sized private facilities, have had shorter stays, more frequent external visits, fewer chronic conditions, and lower degrees of functional dependency at baseline than those with low or moderate levels of SE (Table 1).

**Table 1:** Baseline characteristics of residents by level of social engagement in nursing homes in Madrid, Spain^a^

Characteristic	Overall	Level of social engagement			***P*** value^b^
		Low/null	Moderate	High	
No. of residents	382 (100)	124 (36.0)	179 (44.2)	79 (19.8)	
Age (years)					0.03
65–74	61 (15.7)	15 (13.3)	27 (11.6)	19 (29.4)	
75–79	70 (16.4)	21 (17.3)	33 (15.1)	16 (17.4)	
80–84	102 (25.6)	31 (21.2)	50 (30.0)	21 (24.1)	
85–89	81 (23.8)	37 (30.6)	34 (22.8)	10 (13.4)	
≥ 90	68 (18.5)	20 (17.6)	35 (20.5)	13 (15.7)	
Sex					0.01
Women	200 (74.4)	79 (82.8)	87 (70.9)	34 (66.9)	
Men	182 (25.6)	45 (17.2)	92 (29.1)	45 (33.1)	
Educational level					0.78
Less than primary	188 (45.3)	60 (41.8)	90 (49.0)	38 (43.3)	
Primary	143 (40.2)	49 (44.3)	66 (36.9)	28 (39.9)	
Secondary or more	51 (14.6)	15 (13.9)	23 (14.1)	13 (16.8)	
Marital status					0.35
Married	68 (14.2)	16 (11.3)	36 (15.3)	16 (17.1)	
Single	113 (33.7)	44 (40.6)	51 (30.9)	18 (27.3)	
Widowed/divorced	201 (52.1)	64 (48.1)	92 (53.8)	45 (55.5)	
Facility ownership					0.10
Public/subsidized	285 (61.4)	89 (57.7)	142 (68.2)	54 (52.7)	
Private	97 (38.6)	35 (42.3)	37 (31.8)	25 (47.3)	
Facility size (beds)					0.15
< 100	51 (20.8)	18 (21.8)	22 (20.3)	11 (20.0)	
100–299	138 (37.1)	47 (38.9)	56 (30.6)	35 (48.4)	
≥ 300	193 (42.1)	59 (39.3)	101 (49.1)	33 (31.6)	
Length of stay (years)					0.01
0–1	117 (29.0)	29 (22.3)	56 (28.6)	32 (42.2)	
2–4	122 (31.9)	40 (32.4)	54 (29.0)	28 (37.3)	
≥ 5	143 (39.1)	55 (45.3)	69 (42.4)	19 (20.4)	
Assigned caregiver					0.47
Yes	57 (13.6)	18 (11.6)	29 (16.1)	10 (11.5)	
No	325 (86.4)	106 (88.4)	150 (83.9)	69 (88.5)	
Frequency of external visits					0.22
Monthly or less	144 (33.6)	54 (37.9)	71 (35.0)	19 (22.7)	
Weekly	168 (45.4)	46 (39.0)	80 (47.2)	42 (53.0)	
Daily	70 (21.0)	24 (23.2)	28 (17.8)	18 (24.3)	
No. of chronic conditions					0.27
0–1	109 (30.0)	33 (32.0)	44 (24.6)	32 (38.6)	
2–3	174 (45.9)	57 (42.6)	84 (49.4)	33 (44.1)	
≥ 4	99 (24.1)	34 (25.4)	51 (26.0)	14 (17.3)	
Functional dependency					0.08
No	152 (35.0)	40 (29.7)	69 (33.4)	43 (48.2)	
Mild	128 (34.5)	41 (33.3)	67 (39.4)	20 (26.0)	
Moderate	102 (30.5)	43 (37.0)	43 (27.3)	16 (25.8)	

^a^Unweighted counts (sampling-weighted percentages)

^b^*P* value for homogeneity of sampling-weighted percentages across levels of social engagement

**Fig. 1:**
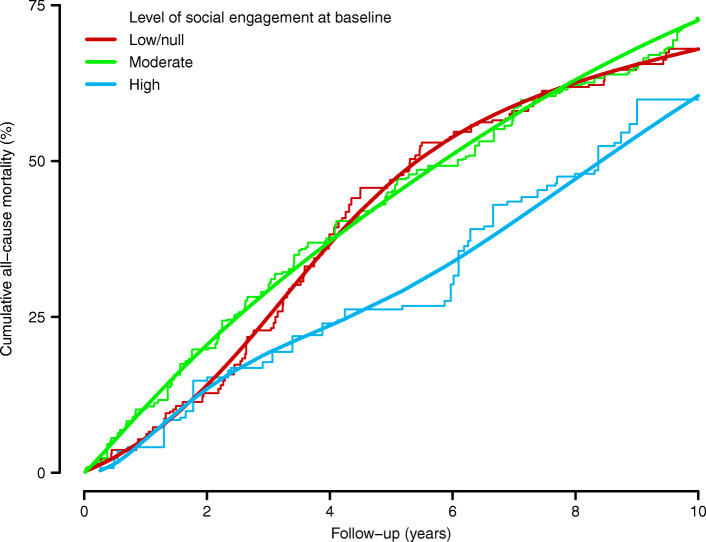
Standardized cumulative all-cause mortality by level of social engagement at baseline among residents in nursing homes of Madrid, Spain, 1998–1999 to 2009. Parametric cumulative mortality curves (smooth lines) were estimated from a spline-based survival model and nonparametric cumulative mortality curves (step functions) from Kaplan-Meier methods, both weighted by combined inverse probability weights and stratified by baseline level of social engagement. Combined weights were used to standardize cumulative mortality curves in each level of social engagement to the weighted distribution of baseline confounders in the overall institutionalized population, including age (65–74, 75–79, 80–84, 85–89, or ≥ 90 years), sex (women or men), educational level (less than primary, primary, or secondary or more), marital status (married, single, or widowed/divorced), facility ownership (public/subsidized or private), facility size (< 100, 100–299, or ≥ 300 beds), length of stay (0–1, 2–4, or ≥ 5 years), assigned caregiver (yes or no), frequency of external visits (monthly or less, weekly, or daily), number of chronic conditions (0–1, 2–3, or ≥ 4), and functional dependency (no, mild, or moderate)

A total of 268 participants died during 2305 person-years of follow-up (median follow-up 6.2 years), with an overall mortality rate of 10.9 deaths per 100 person-years. All-cause mortality was consistently lower after the first 2 years of follow-up among residents with high levels of SE at baseline, compared to those with low/null or moderate levels (Fig. 1**);** this was after standardization to the overall weighted population distribution of sociodemographic characteristics, facility features, multimorbidity, and disability at baseline. Compared with residents with low/null levels of SE, the standardized mortality risk differences (95% CIs) at 5 and 10 years of follow-up were − 2.3% (− 14.6 to 10.0%) and 4.6% (− 7.4 to 16.6%) for moderately engaged residents; and − 18.4% (− 33.8 to − 2.9%) and − 7.5% (− 24.2 to 9.3%) for highly engaged residents (Table 2, Model 3). Similarly, the fully-standardized differences (95% CIs) in the median survival time when comparing residents with low/null SE at baseline were 0.4 (− 1.4 to 2.2) years for moderate SE and 3.0 (0.8 to 5.2) for high SE.

**Table 2:** Standardized differences in cumulative all-cause mortality at 2, 5, and 10 years of follow-up by level of social engagement at baseline among residents in nursing homes in Madrid, Spain

	Level of social engagement at baseline		
	Low/null	Moderate	High
No. of person-years	691.1	1042.2	571.6
No. of deaths	92	133	43
Mortality rate^a^	11.6	12.8	6.6
**2-year follow-up**			
Cumulative mortality^b^ (%)	11.8	18.8	8.1
Standardized risk difference^c^ (95% CI)			
Model 1^d^	0.0 (reference)	3.9 (−4.4 to 12.1)	−6.5 (−15.0 to 1.9)
Model 2^e^	0.0 (reference)	5.0 (−3.5 to 13.4)	−2.3 (−13.9 to 9.3)
Model 3^f^	0.0 (reference)	6.5 (−2.3 to 15.4)	−0.6 (−11.8 to 10.7)
**5-year follow-up**			
Cumulative mortality^b^ (%)	47.4	46.2	20.1
Standardized risk difference^c^ (95% CI)			
Model 1^d^	0.0 (reference)	−3.7 (−15.6 to 8.2)	−24.3 (− 37.4 to −11.1)
Model 2^e^	0.0 (reference)	−4.5 (−16.7 to 7.8)	−22.5 (−38.2 to −6.7)
Model 3^f^	0.0 (reference)	−2.3 (−14.6 to 10.0)	−18.4 (−33.8 to −2.9)
**10-year follow-up**			
Cumulative mortality^b^ (%)	68.9	74.4	50.5
Standardized risk difference^c^ (95% CI)			
Model 1^d^	0.0 (reference)	4.3 (−7.3 to 15.9)	−9.4 (−25.8 to 7.1)
Model 2^e^	0.0 (reference)	3.8 (−7.7 to 15.3)	−9.3 (−26.0 to 7.4)
Model 3^f^	0.0 (reference)	4.6 (−7.4 to 16.6)	−7.5 (−24.2 to 9.3)

^a^ Sampling-weighted mortality rates per 100 person-years

^b^Unstandardized cumulative mortality risks at the specified follow-up times were obtained from sampling-weighted Kaplan-Meier methods stratified by level of social engagement at baseline

^c^ Standardized differences in cumulative mortality at the specified follow-up times which compared levels of social engagement at baseline were obtained from spline-based survival models weighted by combined inverse probability weights and stratified by level of social engagement. 95% confidence intervals (CIs) were derived from robust standard errors of spline coefficients by applying delta methods

^d^ Model 1 was standardized for baseline age (65–74, 75–79, 80–84, 85–89, or ≥ 90 years), sex (women or men), educational level (less than primary, primary, or secondary or more), and marital status (married, single, or widowed/divorced)

^e^ Model 2 was further standardized for baseline facility ownership (public/subsidized or private), facility size (< 100, 100–299, or ≥ 300 beds), length of stay (0–1, 2–4, or ≥ 5 years), assigned caregiver (yes or no), and frequency of external visits (monthly or less, weekly, or daily)

^f^ Model 3 was further standardized for baseline number of chronic conditions (0–1, 2–3, or ≥ 4) and functional dependency (no, mild, or moderate)

**Fig. 2:**
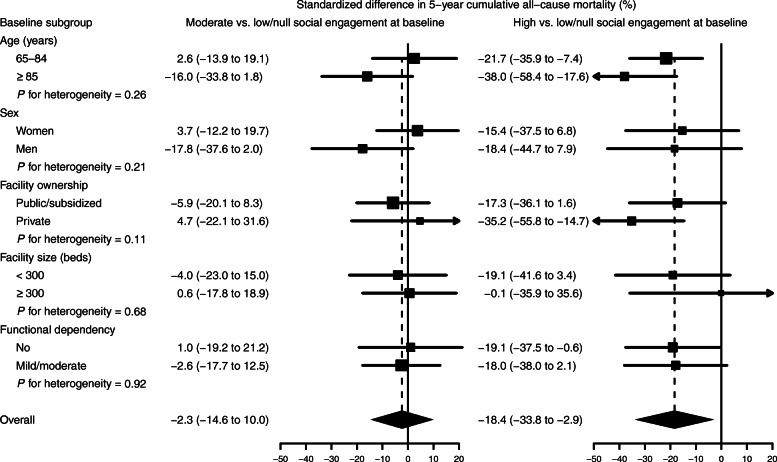
Standardized differences in 5-year cumulative all-cause mortality comparing moderate and high with low/null social engagement at baseline in pre-specified subgroups of residents in nursing homes in Madrid, Spain, 1998–1999 to 2009. Subgroup-specific risk differences (squares with area inversely proportional to the variance) and their 95% confidence intervals (horizontal lines) were obtained from spline-based survival models weighted by combined inverse probability weights and stratified by baseline level of social engagement and resident subgroup. Subgroup-specific weights were used to standardize cumulative mortality in each social engagement level and resident subgroup to the weighted distribution of baseline confounders in the entire resident subgroup, including age (65–74, 75–79, 80–84, 85–89, or ≥ 90 years), sex (women or men), educational level (less than primary; primary; or secondary or more), marital status (married, single, or widowed/divorced), facility ownership (public/subsidized or private), facility size (< 100, 100–299, or ≥ 300 beds), length of stay (0–1, 2–4, or ≥ 5 years), assigned caregiver (yes or no), frequency of external visits (monthly or less, weekly, or daily), number of chronic conditions (0–1, 2–3, or ≥ 4), and functional dependency (no, mild, or moderate)

In subgroup analyses, the standardized differences in 5-year mortality risk for moderate versus low/null SE at baseline reached − 16.0% in residents aged 85 years or older and − 17.8% in men, whereas the standardized 5-year risk differences for high versus low/null SE increased to − 38.0% in older residents and − 35.2% in private facilities (Fig. 2). Nevertheless, no significant heterogeneity in risk differences was detected across any resident subgroup and the limited sample size resulted in imprecise estimates in some subgroups.

## Discussion

Residents with high SE levels within the nursing home had better 5-year overall survival rates when compared with residents with low/null levels of SE. Furthermore, this association was independent of baseline sociodemographic characteristics, facility features, multimorbidity, disability and frequency of contact with people outside the nursing home. Moreover, residents with high internal SE had a 3-year increase in the median survival time when compared with residents with low/null SE. We also found that this benefit in 5-year overall survival was more notable for over-85 s and those in private facilities.

SE encompasses a great variety of activities, most of which have consistently been shown to be associated with better physical and mental health [2]. In addition, the hypothesis supported by our results – greater SE improving survival – has biological plausibility. Perceived social isolation in humans seems to affect physiological responses, with adverse chronic consequences like activation of the hypothalamic-pituitary-adrenal axis, glucocorticoid insensitivity, alteration of the immune system [20] and increased risk for inflammatory disease [21]. Inflammation and oxidation generate a vicious circle that damages protein, carbohydrates, lipids, and DNA and its repair [22, 23] and predisposes to many chronic disorders [23].

As far as we know, only three recent studies carried out with nursing home residents have assessed the relation between SE and mortality over follow-up periods longer than 1 year. Hjaltadottir et al.’s multicenter study found an increased 3-year mortality risk in residents with lower SE compared to those with high SE, but adjustment was limited to just age and gender [10]. In a study based on a large facility, Kiely et al. reported that residents who did not engage socially were 1.4 times more likely to die during a 4-year follow-up period, when compared with the most socially engaged residents and after adjustment for the main confounders [8]. Cohen-Mansfield et al. in an 11-year follow-up study in a nursing home, found a positive association between social network quality and survival, but this relation weakened after adjustment for the main confounders [9].

It is worth noting some of the limitations of the current study. First, as this study was a secondary analysis of data, we did not use a standardized scale to evaluate SE; it was estimated through a specific question about the degree of interaction with other residents in the facility and degree of active participation in facility activities, with the responses assigned to a 3-category scale. Second, SE and the main covariates were only measured at baseline, so we cannot exclude that those variables may have changed over the follow-up period, and that modification could have influenced the outcome. Third, some deaths may have not been recorded. This possible limitation would have probably generated a non-differential misclassification and potentially attenuated the associations found. Fourth, we did not adjust for frailty, which could be associated with both SE and mortality. However, we did control for functional dependency and comorbidities among other co-variates, which could collectively serve as effective proxies for frailty and other potential confounders. This suggests that our results are robust. Furthermore, our study design and statistical procedures embraced the basic elements of an intervention study, like exclusion criteria – which limited confounding by restricting important confounding categories – and inverse probability weighting – which tries to emulate a random assignment of study participants to each of the study groups. All of this would have reduced confounding to a minimum. Thus, assuming a limited influence of unmeasured confounding, this design might be assimilated to an intervention trial of a hypothetical SE program with perfect compliance. Other strengths worth mentioning are that our study used a probabilistic sample of the nursing home population, with a high response rate and long follow-up, and that measurements were obtained directly by trained interviewers. The representativeness of our sample makes reasonable to consider that our results, relating high SE with a better survival, could be extrapolated to nursing homes in many other geographical areas. In fact, our results are consistent with some studies that analyzed this association in single facilities from other geographic locations [8, 9].

## Conclusions

Residents with high SE within the nursing home had lower mortality risk when compared with residents with low/null SE. This association was independent of sociodemographic characteristics, facility features, comorbidities, disability and frequency of contact with people outside the nursing home.

Participation in varied activities within the facility has been shown to be very beneficial for physical and mental health, and our study provides convincing results regarding survival for people living in care homes. Furthermore, what is important is that this increase in life expectancy also occurs in a way that surely improves quality of life, as opposed to life prolongation through aggressive treatment strategies – which may increase survival but at the cost of undermining quality of life. Thus, our results suggest that it would be advisable for the clinical practice in nursing home settings to include adequate interventions, in line with the ones analyzed by Grenade et al. [5], which have shown to be effective in increasing SE in nursing home residents.

However, to implement the adequate activities that increase the SE of the residents requires the facility managers and staff to be proactive not only by putting in place the adequate activities that encourage personal contact, but also by fighting against the impediments which are habitual in such institutions. This may be because many residents are very passive or reticent, or the staff has an excessive workload, among other barriers [24].

## Supplementary Information

Additional file 1: **Supplementary Table 1**. Distribution of baseline characteristics by level of social engagement after standardization to the overall institutionalized population in Madrid, Spain. **Supplementary Figure 1**. Distribution of combined weights taking into account both sampling and standardization based on the three polytomous logistic models for baseline social engagement in care homes in Madrid, Spain.

Additional file 2 English translation of the relevant questions used in this work.

## Data Availability

The dataset supporting the conclusions of this article is available in the Repisalud repository, http://hdl.handle.net/20.500.12105/11202

## Abbreviations

SE: Social engagement
CI: Confidence interval
