# Supplementary material for: Social engagement within the facility increased life expectancy in nursing home residents: a follow-up study
Source: BMC Geriatr. 2020 Nov 18;20:480. doi: 10.1186/s12877-020-01876-2 (PMC7672974; doi:10.1186/s12877-020-01876-2)
Supplement: Supplementary file 1 — Supplementary Table 1. Distribution of baseline characteristics by level of social engagement after standardization to the overall institutionalized population in Madrid, Spain. Supplementary Figure 1. Distribution of combined weights taking into account both sampling and standardization based on the three polytomous logistic models for baseline social engagement in care homes in Madrid, Spain. [file 12877_2020_1876_MOESM1_ESM.pdf]

Supplementary Table 1. Distribution of baseline characteristics by level of social engagement after standardization to the overall institutionalized population in Madrid, Spain.

		Level of social engagement†			
**Characteristic**	**Overall***	**Low/null**	**Moderate**	**High**	***P* value‡**
Age (years)					1.00
65–74	15.7	14.7	14.9	18.1	
75–79	16.4	15.0	15.8	15.2	
80–84	25.6	28.8	27.3	29.4	
85–89	23.8	24.1	23.3	21.3	
≥90	18.5	17.6	18.7	16.0	
Sex					0.90
Women	74.4	73.2	71.8	70.2	
Men	25.6	26.8	28.2	29.8	
Educational level					0.99
Less than primary	45.3	47.8	46.4	45.8	
Primary	40.2	40.0	39.5	42.1	
Secondary or more	14.6	12.2	14.2	12.1	
Marital status					0.90
Married	14.2	13.1	14.3	17.7	
Single	33.7	31.5	32.8	26.3	
Widowed/divorced	52.1	55.4	53.0	56.0	
Facility ownership					0.99
Public/subsidized	61.4	63.8	63.4	64.2	
Private	38.6	36.2	36.6	35.8	
Facility size (beds)					0.87
<100	20.8	19.2	19.6	16.3	
100–299	37.1	38.5	36.7	46.0	
≥300	42.1	42.3	43.7	37.7	
Length of stay (years)					0.89
0–1	29.0	30.4	29.3	32.5	
2–4	31.9	31.5	32.1	36.9	
≥5	39.1	38.1	38.5	30.5	
Assigned caregiver					0.72
No	86.4	87.9	85.0	83.4	
Yes	13.6	12.1	15.0	16.6	
Frequency of external visits					0.99
Monthly or less	33.6	34.5	36.3	33.5	
Weekly	45.4	45.1	44.9	44.1	
Daily	21.0	20.4	18.8	22.4	
No. of chronic conditions					0.71
0–1	30.0	29.1	29.2	39.7	
2–3	45.9	47.3	47.3	40.3	
≥4	24.1	23.6	23.4	20.0	
Functional dependency					0.95
No	35.0	34.3	34.5	38.5	
Mild	34.5	34.9	35.0	28.8	
Moderate	30.5	30.8	30.5	32.7	

* Sampling-weighted percentages.
† Fully-weighted percentages taking into account both sampling and standardization weights.
‡ *P* value for homogeneity of fully-weighted percentages across levels of social engagement.

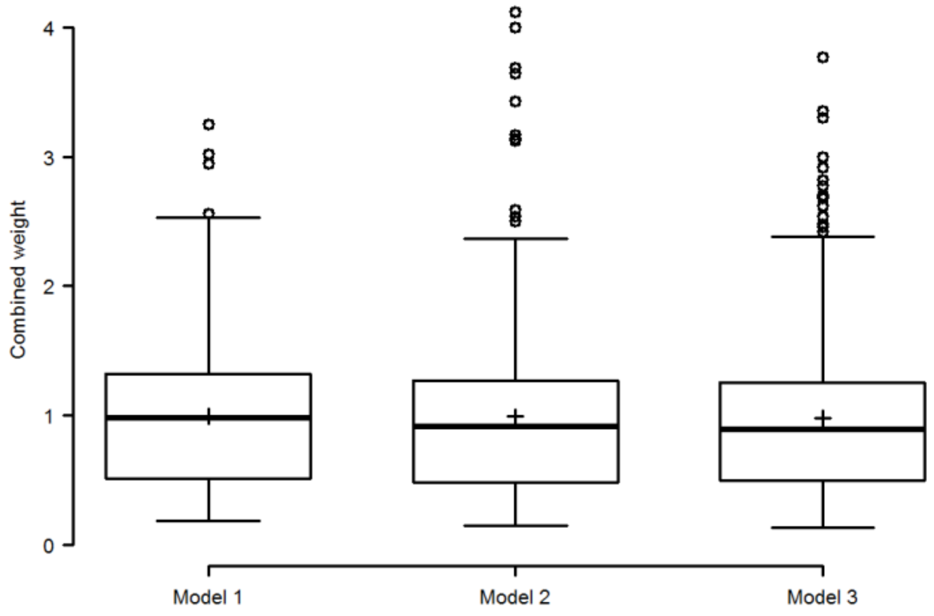

Supplementary Figure 1. Distribution of combined weights taking into account both sampling and standardization based on the three polytomous logistic models for baseline social engagement in care homes in Madrid, Spain.

Boxes represent the mean (+), median (middle horizontal line), quartiles (border horizontal lines), and individual outlying weights (circles).
