# Supplementary material for: Social engagement within the facility increased life expectancy in nursing home residents: a follow-up study
Source: BMC Geriatr. 2020 Nov 18;20:480. doi: 10.1186/s12877-020-01876-2 (PMC7672974; doi:10.1186/s12877-020-01876-2)
Supplement: Supplementary file 2 — English translation of the relevant questions used in this work. [file 12877_2020_1876_MOESM2_ESM.pdf]

ENGLISH VERSION OF THE RELEVANT QUESTIONS USED IN THIS WORK

SOCIODEMOGRAPHIC

1. Age, in completed years

2. Sex 1 Man 2 Women.

3. Date of entry into the facility

4. Civil status

1 Married
2 Single
3 Widow/Widower
4 Separated or divorced
9 Don´t know / does not answer.

5. Formal education

1 Cannot read or write
2 Can read and write
3 Primary education
4 Secondary education
5 University education
9 Don´t know / does not answer.

Answers: 1. Caregiver 2. Resident

RESIDENT COGNITIVE (MENTAL) STATUS
[MDS COGNITION SCALE ITEMS]

6. Does the resident have problems of short-term memory?
1. Yes 2. No 9. Don't know.

7. Does the resident have long-term memory problems?
1. Yes 2. No 9. Don't know.

8. Is the resident able to recall his/her own room?
1. Yes 2. No 9. Don't know.

9. Does the resident know that s/he is in a nursing home?
1. Yes 2. No 9. Don't know.

10. Is the resident normally able to recall staff names/faces?
1. Yes 2. No 9. Don't know.

11. Is the resident normally able to recall the current season?
1. Yes 2. No 9. Don't know

12. Respect to the resident's capacity for making decisions regarding tasks of daily life, the resident is

1 INDEPENDENT. Decisions are consistent/reasonable
2 SOME DIFFICULTY, but just in new situations only
3 DECISIONS POOR. Needs supervision to make any day-to-day decision
4 SEVERELY IMPAIRED. Never/rarely makes decisions.

13. Respect to the resident's capacity for making self-understood, the resident…

1 NO DIFFICULTY. S/he makes her/himself understood
2 MILD DIFFICULTY. S/he generally makes her/himself understood even though s/he has difficulty finding a word or finishing a sentence
3 MODERATE DIFFICULTY. S/he makes her/himself understood sometimes, but her/his ability is limited to ask for specific things
4 SEVERE DIFFICULTY. S/he rarely makes her/himself understood.

14. Resident's capacity for independent dressing

1 Independent
2 Dressed by her/himself but needs supervision
3 Needs some active help
4 Needs important active help
5 Total dependence for dressing
9 Don't know.

15. PFEIFFER'S QUESTIONNAIRE

1 What date is today?
2 What day of the week is today?
3 What is this city called?
4 What is your room number?
5 How old are you?
6 What is your birth date?
7 What is the name of the king of Spain?
8 What was the name of the former head of the Spanish state?
9 What is your mother's second last name?
Subtract 3 from 20 and do the same with each number you get until you can no longer subtract.

Instructions
Ask questions 1 to 10 and write down all the answers. A question is correct only if all its parts are correct. For example: if a subject gives the correct month and year when asked for the date, but misses the day, this question is considered wrongly answered. The name of the city, town, neighborhood, parish or place is accepted as correct answers to question 3. Record the total number of errors based on the ten questions.

Interpretation for people with primary education
0-3 errors: normal intellectual functioning.
4-5 errors: mild or doubtful intellectual impairment.

6-8 errors: moderate intellectual impairment.
9-10 errors: major intellectual impairment.

Interpreting for people with other levels of education
Allow one more error if the person does not read or write, one less error if they have received secondary education, and two fewer errors if they have received higher education.

* Cross-cultural adaptation to Spanish society. Grau Fibla G, Eiroa Patiño P, Cayuela Domínguez A. Spanish version of the OARS Multidimensional Functional Assessment Questionnaire. Technical document of the Andalusian Health Council. Directorate General for Coordination, Teaching and Research.
** Note from the Department. Original Question 4: What is your phone number? and question 4.a (only if the person does not have a telephone): What is their address? It has been substituted for the residential environment. Record the total number of errors. Unanswered questions are considered errors.

FAMILY AND FRIENDS [social engagement]

16. The resident interacts with the other residents of the institution

1 A lot; actively engages in the facility activities
2 Sufficiently, normal
3 Hardly
4 Not at all.

17. The resident interacts with family and friends outside the institution (telephone or personal contact)

1 A lot (daily)
2 Regularly (weekly contact)
3 Infrequently (monthly contact)
4 Very infrequently (less than a monthly contact)
5 Not at all
9 Don't know.

BARTHEL INDEX. Check the option that best suits you regarding the following self-care activities. Please indicate what the resident does independently, not what they can or could do in a hypothetical case.

18. FEEDING in the last 7 days

1 TOTALLY INDEPENDENT.
2 NEEDS MINIMUM HELP. S/he is independent if a tray is prepared for her/him, but s/he needs help cutting the meat, opening a milk carton or opening a jar of jam. Otherwise the caregiver may be absent.
3 MODERATE HELP. Eats alone but needs supervision throughout the meal, needs help with tasks like putting sugar, salt or pepper, or spreading butter.
4 GREAT HELP. Can use some eating device, usually a spoon, but someone must provide assistance during the meal.
5 DEPENDENT in all aspects.

19. PERSONAL HYGIENE in the last 7 days

1 TOTALLY INDEPENDENT. It includes washing face and hands, comb hair, clean teeth and shaving using the razor plug by himself if it is electric.
2 NEEDS MINIMUM HELP in some of the above, (e.g.: plugging / unplugging the razor), but runs the entire process alone.
3 MODERATE HELP needs help with one or more of the above tasks (ex: washing and shaving, even combing by her/himself)
4 BIG HELP requires assistance in all steps of hygiene, but helps a bit.
5 DEPENDENT in all respects.

20. DRESSING in the last 7 days

1 TOTALLY INDEPENDENT, includes fastening and unbuttoning clothing and buttons and tying / untying shoelaces
2 NEEDS MINIMUM HELP for what is specified above (a button or shoelace), but the resident already puts on and removing alone all the clothes or shoes
3 MODERATE HELP, needs assistance in putting on or removing any item (clothing or shoes)
4 GREAT HELP the resident participates to some degree but is dependent on all aspects of clothing
5 DEPENDENT in all aspects, does not participate in the activity.

21. BATHING in the last 7 days

1 TOTALLY INDEPENDENT, does not need any other person present. It can use the bathtub, shower, or when washing body parts including the back.
2 NEEDS MINIMUM HELP. Requires supervision for safety, to enter / exit the bathtub, adjusting the water temperature, etc.
3 MODERATE HELP, requires assistance to get in / out of the bathtub, wash or dry off.
4 GREAT HELP, needs assistance in all phases of the bath, but helps a bit
5 DEPENDENT in all aspects, does not collaborate in the activity.

22. BOWELS in the last 7 days

1 CONTINENT / INDEPENDENT. It is continent of bowel and independent to use suppositories or enemas
2 INCONTINENT OCCASIONAL / MINIMAL HELP. Occasional fecal incontinence accidents. Requires supervision for suppositories or enemas, but is given by her/himself
3 INCONTINENT FREQUENT / MODERATE HELP. Frequent accidents, but can assume appropriate position for bowel movements and enemas. Requires someone to give him suppositories and enemas. Requires help putting on pads.
4 INCONTINENT / GREAT HELP. Almost continuous accidents. The resident needs help assuming the appropriate position, but collaborates to some extent.
5 TOTAL INCONTINENT / TOTAL DEPENDENT.

23. BLADDER in the last 7 days

1 CONTINENT / INDEPENDENT. Continent day and night. Independent to handle catheter, collection bag, etc.
2 INCONTINENT OCCASIONAL / MINIMAL HELP. Generally dry day and night, but can have occasional accident. Needs minimal assistance with catheter or diaper.
3 INCONTINENT FREQUENT / MODERATE HELP. Generally dry during the day but not at night. Needs assistance with catheter or diaper.
4 INCONTINENT / GREAT HELP. Incontinent. Collaborates in the placement of the catheter or diaper.

5 TOTAL INCONTINENT / TOTAL DEPENDENT

24. ON AND OFF THE TOILET in the last 7 days

1 TOTALLY INDEPENDENT. Is able to get on and off the toilet by himself, fasten and unfasten clothes, puts them in place, avoids staining clothes and uses toilet paper without help. Can use a urinal at night but must be able to empty and clean it.
2 NEEDS MINIMUM HELP. Requires supervision for safety. Requires assistance to empty and clean the urinal.
3 MODERATE HELP. Requires active assistance for some phase, such as handling clothes, getting up or sitting down, or washing hands.
4 GREAT HELP. Requires assistance in all phases, but collaborates
5 DEPENDENT in all aspects, does not participate in the activity.

25. CHAIR/BED TRANFERS in the last 7 days

1 TOTALLY INDEPENDENT to get up and go to bed. In the case of a patient in a wheelchair, s/he can approach the bed in the wheelchair, brake it, raise the footrests, get into bed, lie down, sit on the edge of the bed again, change the position of the wheelchair and sit back in it.
2 NEEDS MINIMUM HELP. Requires supervision for safety, but gets up and goes to bed by her/himself.
3 MODERATE HELP. Requires minimal assistance from a not very strong person to lie down or get out of bed.
4 GREAT HELP. Needs maximum assistance from another person, but collaborates in the activity.
5 DEPENDENT. Two people are needed to the chair-bed transfer. Unable to collaborate.

26. STAIRS in the last 7 days

1 TOTALLY INDEPENDENT. Walks up and down a flight of stairs without help and supervision. Can use a handrail, cane or crutch and must carry them up the stairs regardless of whether s/he uses them.
2 NEEDS MINIMUM HELP. Usually does not require assistance although sometimes requires supervision for safety, for example for morning stiffness, shortness of breath, etc.
3 MODERATE HELP. Needs some assistance or does it alone but without being able to carry the aids s/he normally uses.
4 GREAT HELP. Requires assistance in all aspects, but s/he collaborate
5 DEPENDENT. Totally dependent, does not go up or down stairs.

27. AMBULATION in the last 7 days. IF THE RESIDENT USES A WHEELCHAIR, DO NOT ANSWER THIS QUESTION AND GO TO NEXT QUESTION

1 TOTALLY INDEPENDENT. Walks 50 meters without help or supervision. S/he can use any aid (cane, walker, crutch), and has no problem reaching and manipulating them.
2 NEEDS MINIMUM HELP. Walks alone, but not as long as 50 meters without help or supervision. Need to be supervised in dangerous situations.
3 MODERATE HELP. Requires assistance to reach the aids (e.g.: someone have to give or put the cane or walker in front of her/him) or maneuver them (e.g.: open the walker if it is foldable). Or it requires the assistance of a person even for short distances.
4 GREAT HELP. It requires the presence of two or more people during the walking.
5 DEPENDENT. Unable, or does not walk at all.

28. RESIDENT WITH WHEELCHAIR in the last 7 days

1 TOTALLY INDEPENDENT. Able to propel the chair at least 50 meters by itself, go around corners, turn around, steer and position it properly next to the table, bed and toilet.
2 NEEDS MINIMUM HELP. Self-propelled, but needs minimal help in tight corners.
3 MODERATE HELP. A person is needed to position the chair in the proper position beside the bed, on the table, manipulate the chair in the room, etc.
4 GREAT HELP. Self-propelled only short distances on flat ground
5 DEPENDENT. Independent; needs someone else to the chair.

QUESTIONS TO THE PHYSICIAN OR NURSE

Answers 1. Physician 2. Nurse.

DISEASES

29. Has the resident suffered an acute stroke in the past year?
1. Yes 2. No 9. Don’t know.

30. Indicate if the resident has (or does not have) any of these diseases:

Cardiac arrhythmia	1 Yes 2 No
Congestive heart failure	1 Yes 2 No
Ischemic heart disease	1 Yes 2 No
Hypertension	1 Yes 2 No
Leg circulatory disease	1 Yes 2 No
Emphysema, asthma, chronic bronchitis	1 Yes 2 No
Alzheimer's dementia	1 Yes 2 No
Dementia of another type	1 Yes 2 No
Parkinson's disease or parkinsonism	1 Yes 2 No
Anxiety disorder	1 Yes 2 No
Depression	1 Yes 2 No
Anemia	1 Yes 2 No
Cancer	1 Yes 2 No
Diabetes	1 Yes 2 No
Arthritis or severe osteoarthritis	1 Yes 2 No
Epilepsy or seizure	1 Yes 2 No
